# Salt-induced transcription factor *MYB74* is regulated by the RNA-directed DNA methylation pathway in *Arabidopsis*

**DOI:** 10.1093/jxb/erv312

**Published:** 2015-07-02

**Authors:** Rui Xu, Yuhan Wang, Hao Zheng, Wei Lu, Changai Wu, Jinguang Huang, Kang Yan, Guodong Yang, Chengchao Zheng

**Affiliations:** ^1^State Key Laboratory of Crop Biology, College of Life Sciences, Shandong Agricultural University, Taian, Shandong 271018, PR China; ^2^Shandong Provincial Hospital Affiliated to Shandong University, Jinan, Shandong 250021, PR China

**Keywords:** *Arabidopsis*, *AtMYB74*, RNA-directed DNA methylation, salt stress, siRNA, transcription, transcription factor.

## Abstract

Salt stress is one of the major abiotic stresses in agriculture worldwide that causes crop failure by interfering with the profile of gene expression and cell metabolism. Transcription factors and RNA-directed DNA methylation (RdDM) play an important role in the regulation of gene activation under abiotic stress in plants. This work characterized *AtMYB74*, a member of the R2R3-MYB gene family, which is transcriptionally regulated mainly by RdDM as a response in salt stress in *Arabidopsis*. Bisulphite sequencing indicated that 24-nt siRNAs target a region approximately 500bp upstream of the transcription initiation site of *AtMYB74*, which is heavily methylated. Levels of DNA methylation in this region were significantly reduced in wild type plants under salt stress, whereas no changes were found in RdDM mutants. Northern blot and quantitative real-time reverse transcription PCR analysis showed that the accumulation of 24-nt siRNAs was decreased in WT plants under salt stress. Further promoter deletion analysis revealed that the siRNA target region is essential for maintaining *AtMYB74* expression patterns. In addition, transgenic plants overexpressing *AtMYB74* displayed hypersensitivity to NaCl during seed germination. These results suggest that changes in the levels of the five 24-nt siRNAs regulate the *AtMYB74* transcription factor via RdDM in response to salt stress.

## Highlight

*AtMYB74*, a R2R3-MYB gene, is transcriptionally regulated through RdDM for response to salt stress. The accumulation of siRNA targeting to the *AtMYB74* promoter region is essential for maintaining *AtMYB74* expression.

## Introduction

High salinity is a crucial problem affecting plant growth and crop production in many parts of the world. Recently, many salt-stress responsive genes and protein have been identified by both forward and reverse genetics approaches. The transcription factors, such as CCAAT-binding transcription factors (CBFs), NAM, ATAF1/2 CUC2 transcription factors (NACs), and WRKYs, act as key regulators in response to salt stress in plants (Singh *et al.*, 2002). The MYB proteins also function as transcription factors that play regulatory roles in the defence responses of plants (Jin and Martin, 1999; Zheng *et al.*, 2012). R2R3-MYB is the largest subfamily of the MYB family, which includes 126 members and can be further categorized into 22 subgroups (Stracke *et al.*, 2001). To date, several R2R3-MYB proteins have been reported to be involved in the abiotic stress responses of *Arabidopsis*. Overexpression of *AtMYB2* results in the enhanced expression of *RD*22 (a dehydration-responsive gene) and *AtADH1* (alcohol dehydrogenase1), and the transgenic plants display higher sensitivity to abscisic acid (Abe *et al.*, 2003). The mutants of *AtMYB108* are hypersensitive to salt, drought, and oxidative stresses, which are possibly mediated by reactive oxygen intermediates (Mengiste *et al.*, 2003). Subgroup 11 consists of three members, *AtMYB41*, *AtMYB102*, and *AtMYB74*. *AtMYB41* is involved in the control of primary metabolism and negative regulation of short-term transcriptional responses to osmotic stress (Lippold *et al.*, 2009). *AtMYB102* functions as a key factor in both osmotic stress and wounding signalling pathways in *Arabidopsis* (Denekamp and Smeekens, 2003). *AtMYB74*, as one of the stress-upregulated genes, has been reported in a general profile of the expression pattern of the MYB family (Kranz *et al.*, 1998). However, the molecular mechanism of the response of *AtMYB74* to abiotic stress is largely unknown.

Genome expression is mainly influenced by chromatin structure, which is governed by processes often associated with epigenetic regulation, including histone post-translational modification and DNA methylation (Bender, 2004; Zhang, 2008). In histone modification, arginine and lysine methylation is also involved in transcriptional regulation (Liu *et al.*, 2010). Recent evidence indicates that DNA methylation and siRNA participate in the regulation of gene expression in plants in response to environmental stresses (Chinnusamy and Zhu, 2009; Zhang *et al.*, 2013). DNA methylation occurs in the contexts of CG, CHG, and CHH (where H is adenine, cytosine, or thymine) in plants. Methyltransferase 1 (MET1), chromomethylase 3(CMT3), and domains rearranged methyltransferase (DRM) 2 have been characterized to function as DNA methyltransferases that transfer a methyl group to the cytosine bases of DNA to form 5-methylcytosine (Cao and Jacobsen, 2002; Lindroth *et al.*, 2001; Ronemus *et al.*, 1996). MET1 and CMT3 are mainly in charge of the maintenance of CG and CHG methylation. DRM2 is responsible for *de novo* DNA methylation and exhibits the most prominent role in CHH methylation (Cao *et al.*, 2003).

RNA-directed DNA methylation (RdDM), which was first discovered in viroid-infected tobacco, is an important regulatory phenomenon involved in repressive epigenetic modifications that can trigger transcriptional gene silencing (TGS) (Matzke and Mosher, 2014; Wassenegger *et al.*, 1994). Many key components of the RdDM pathway, such as nuclear RNA polymerase (NRP) D1/NRPE1, RNA-dependent RNA polymerase 2 (RDR2), argonaute 4/6, and dicer-like 3 (DCL3), have been identified, and its molecular mechanism has been established (He *et al.*, 2011; Herr *et al.*, 2005; Li *et al.*, 2006; Wierzbicki *et al.*, 2008; Xie *et al.*, 2004; Zheng *et al.*, 2007). In plants, DNA demethylation depends on four bifunctional 5-methylcytosine glycosylases, repressor of silencing 1 (ROS1), DEMETER, DEMETER-like protein (DML) 2, and DML3 (Choi *et al.*, 2002; Gong *et al.*, 2002; Ortega-Galisteo *et al.*, 2008). ROS1 has been found to counteract the robust RdDM pathway at hundreds of discrete regions across the plant genome together with DML2 and DML3 (Ortega-Galisteo *et al.*, 2008; Penterman *et al.*, 2007). Recent evidence reveals that *Arabidopsis* zinc finger DNA 3′ phosphoesterase is a DNA phosphatase that interacts with ROS1 and functions downstream of ROS1 in one branch of the active DNA demethylation pathway (Martinez-Macias *et al.*, 2012).

The gain or loss of DNA methylation is correlated with a considerable decrease or increase in the corresponding amount of mRNA abundance and with the presence or absence of 24-nt siRNAs at each silenced epiallele (Schmitz *et al.*, 2011). Withdrawal of the inducing siRNA signal could result in active or passive demethylation, which in turn leads to the loss of TGS (Gong *et al.*, 2002; Morales-Ruiz *et al.*, 2006). Approximately one-third of methylated DNA loci in *Arabidopsis* is associated with siRNA clusters (Zhang *et al.*, 2006), implying a primary determinant role of siRNAs in DNA methylation. In plants, the most abundant class of siRNAs includes heterochromatic siRNAs that originate from different sources, such as inverted repeats, pseudogenes, and natural *cis*-antisense transcript pairs. Increasing evidence has shown that the activity of siRNAs could be triggered by various environmental stimuli to affect the targeting chromatin structure. In *Craterostigma plantagineum*, an endogenous siRNA is induced during dehydration, which may contribute to dehydration tolerance (Furini *et al.*, 1997). Salt stress also results in a dramatic change in the accumulation of three siRNAs in wheat seedlings (Yao *et al.*, 2010). In *Arabidopsis*, the 24-nt *SRO5-P5CDH* natural antisense transcript siRNA is involved in the cleavage of *P5CDH* mRNA, which in turn increases proline accumulation under salt stress (Borsani *et al.*, 2005). Exposure of *Arabidopsis* plants to salt, UVC, cold, heat, and flood stresses increases global genome methylation in the progeny, in which Dicer-like protein is involved (Boyko *et al.*, 2010).

With the development of bisulphite sequencing, recent studies have revealed previously uncharted subsets of the epigenome and provided insights into the complex interplay between DNA methylation and transcription (Lister *et al.*, 2008; Zhang *et al.*, 2006). Although the salt stress signal transduction pathway has been intensively studied, whether or not DNA methylation/demethylation is involved in this pathway remains unclear. Despite the fact that salinity stress alters the global DNA methylation level in plants (Ferreira *et al.*, 2015; Wang *et al.*, 2014), the mechanism of how siRNA mediates DNA methylation by RdDM to respond to salt stress also needs to be elucidated. The present study shows that the expression of *AtMYB74* is regulated by RdDM, and the accumulation of 24-nt siRNAs is the major contributor to RdDM. The findings provide a new insight into salt stress regulation in plants and allow a better understanding of the role of siRNAs in controlling the RdDM pathway to regulate gene expression in response to abiotic stress.

## Materials and methods

### Plant growth conditions, seed germination assay, and stress treatment

*Arabidopsis thaliana* (Col-0) was used as the wild type (WT) and the genetic background for transgenic plants in this study. Dry seeds were collected and stored in a dehumidifier cabinet for at least 2 months before the seed germination test was performed. Seeds were stratified and sown on agar plates containing 1% Suc as described previously (Abe *et al.*, 2003) at 4 °C for 2 days and then transferred to 23 °C. *Arabidopsis* seedlings were grown under continuous light (70 μmol·m^–2^·s^–1^) at 23±1 °C. Soil-grown *Arabidopsis* and *Nicotiana benthamiana* plants were grown under a 16h light/8h dark photoperiod at 23±1°C. For the germination assay, at least 100 seeds of each genotype were sterilized and sown on Murashige and Skoog (MS) medium supplemented with or without phytohormones or chemicals. Germination was defined as the first sign of radicle tip emergence and scored daily, and the germination results were calculated based on at least three independent experiments. At 3 days post-germination, the plants of the 5-azacytidine (5-azaC) group were transferred to 50mM 5-azaC. 14-day-old seedlings were subjected to NaCl treatments by transfer to MS liquid medium with 150mM NaCl for durations as indicated, respectively. All these treatments were carried out under a growth condition of 16h light/8h dark at 23 °C unless otherwise mentioned.

### Plasmid constructions

The binary vector pBI121 used for overexpression of *AtMYB74* and the binary vector pFGC5941 used for RNAi were introduced into *Agrobacterium tumefaciens* strain GV3101 and the *Arabidopsis thaliana* (Col-0) plants were transformed by floral dipping. All constructs were verified by sequencing. The transgenic plants were screened on MS medium containing 50 µg ml^–1^ kanamycin for pBI121 and 10 µg ml^–1^ glufosinate ammonium for pFGC5941. T1 transgenic *Arabidopsis* plants were identified by quantitative real-time reverse transcription PCR (qRT-PCR). The corresponding T2 transgenic seedlings that segregated at a ratio of 3:1 (resistant:sensitive) were selected to propagate T3 individuals. RNAi-3 and RNAi-6 were used for further analysis.

### Transient expression in *N. benthamiana*

Different constructs were transformed into *A. tumefaciens* strain GV3101. Overnight cultures were harvested and mixed at a 1:1 ratio with the different construct groups. After incubation for 3h at room temperature in 10mM MgCl_2_, 10mM 2-(*N*-morpholino) ethanesulfonic acid hydrate, pH 5.6, and 150mM acetosyringone, the *Agrobacterium* suspension was coinfiltrated into 3-week-old *N. benthamiana* leaves. Infected leaves were harvested 48h after the infiltration. β-Glucuronidase (GUS) activity and small RNA extraction were performed as described below. Each assay was obtained from at least five independent lines and repeated three times.

### Histochemical GUS staining and fluorometric GUS assay

The promoter sequence of *AtMYB74* was acquired from the TAIR database (http://www.arabidopsis.org/). The promoter–GUS recombinant construct was transformed into *A. tumefaciens* strain GV3101 and then introduced into *Arabidopsis* by the floral dip method. Primers for amplifying the promoter sequence are shown in Supplementary Table S1 at *JXB* online. Histochemical localization of GUS activities in the transgenic seedlings or different tissues was performed after the transgenic plants had been incubated overnight at 37°C in 1mg ml^–1^ 5-bromo-4-chloro-3-indolyl-glucuronic acid, 5mM potassium ferrocyanide, 0.03% Triton X-100 and 0.1M sodium phosphate buffer, pH 7.0. After incubation, the tissues were cleared with 70% ethanol. The cleaned tissues were then observed and photographs were taken by using a stereoscope. For examination of the detailed GUS staining, the tissues were observed with a bright-field microscope and photographed. These GUS staining data were representative of at least five independent transgenic lines for each construct.

Tobacco transgenic plants (100mg) were ground with a mortar with 1000 μl GUS extraction buffer (50mM NaH_2_PO_4_, 10mm EDTA, 0.1% Triton X-100, 0.1% sarcosyl) and centrifuged at 5000rpm for 10min at 4 °C. The crude extract of total protein was obtained from the supernatant. The protein concentration of the extract was determined using a NanoDrop ND-1000 Spectrophotometer (Thermo Fisher Scientific). Fluorometric GUS assays were performed as previously described (Jefferson *et al.*, 1987). GUS activity was measured with 4-methylumbelliferyl-*β*-D-glucuronide as substrate with a Hitachi F-4500 Fluorescence Spectrofluorometer. The standard curves were prepared with 4-methylumbelliferone. GUS activity was expressed as pmol methylumbelliferone min^–1^ mg^–1^ protein. Average GUS activity was obtained from at least five independent transformants and each assay was repeated three times.

### RNA extraction and qRT-PCR analysis

For RNA isolation, leaves and roots of seedlings were harvested separately, frozen in liquid nitrogen, and stored at –80°C until use. Total RNA was isolated from different *Arabidopsis thaliana* seedlings (100mg) with TRIZOL reagent (Invitrogen 15596-026). Contaminated DNA was removed with RNase-free DNase I. First-strand cDNA synthesis was performed with 1 μg RNA using oligo(dT) primer or gene-specific primers. A Prime script RT reagent kit with gDNA Easer (Takara RR047A) was used for all reactions according to the manufacturer’s protocol. cDNAs were diluted 1/50 for qRT-PCR, and qRT-PCR was performed using the FastStart Essential DNA Green Master (Roche 6402712001) and a CFX96 Real-Time System (Bio-Rad). The reaction volume was 15 μl and included 2×FastStart Essential DNA Green Master, 6 μl diluted cDNA, and 1.5 μl of 10 μM primers. Cycle conditions were: 95 °C for 10min; 45 cycles of 95 °C for 10 s, 60 °C for 15 s, and 72 °C for 20 s. Fluorescence was read following each annealing and extension phase. Melt curve analysis of real-time PCR products was performed to verify amplification of a single product. The qRT-PCR experiment was carried out at least three times under identical conditions using tubulin as an internal control. Primers for amplifying genes were designed according to the sequences from the TAIR database (http://www.arabidopsis.org/). Details of primers are listed in Supplementary Table S1. Gene expression was normalized by subtracting the C_T_ value of the control gene from the C_T_ value of the gene of interest. Average expression ratios were obtained from the equation 2^–ΔΔCT^, according to a previously described protocol (Czechowski *et al.*, 2004; Livak and Schmittgen, 2001).

### Northern blot analysis and siRNA qRT-PCR

The RNA blot analysis was carried out as described previously (Yan *et al.*, 2012; Zheng *et al.*, 2007). 50 μg of each RNA was subjected to electrophoresis on a 15% TBE–urea, Criterion gel (Bio-Rad 345-0091) and electroblotted onto Hybond-N+ filter paper (Amersham RPN303B) using a TransBolt-SD apparatus (Bio-Rad 170–3940). The filter then was hybridized at 37 °C in Hybexpression buffer with a ^32^P-labeled probe to detect the five 24-nt siRNAs targeting the *AtMYB74* promoter. The 300bp probe was made by labelling a DNA template that was amplified by primers (listed in Supplementary Table S1) with the Prime-a-Gene Labeling System (Promega U1100) and ^32^P. The filters were washed twice at 37 °C in buffer containing 2×SSC (0.3M NaCl and 0.03M sodium citrate) and 0.5% SDS.

Small RNA for siRNA qRT-PCR was isolated with the miRcute miRNA Isolation kit (TIANGEN DP501), and first-strand cDNA synthesis was performed with the miRcute miRNA first-strand cDNA synthesis kit (TIANGEN KR201-02). siRNA qRT-PCR analysis was performed with 0.5 μg small RNA and a miRcute miRNA qRCR detection kit (TIANGEN FP401). The experiments were performed at least three times under identical conditions using U6 RNA as an internal control (Schmittgen *et al.*, 2004; Yan *et al.*, 2012). Details of primers are listed in Supplementary Table S1.

### Bisulphite sequencing

Aliquots of 800ng DNA were treated with sodium bisulphite using the EZ DNA Methylation-Gold kit (ZYMO RESEARCH D5005) according to the manufacturer’s instructions. The chloroplast genome of every sample was used to calculate the conversion efficiency. Conversion efficiency was >98% for each bisulphite-treated sample. DNA was amplified by PCR with ExTaq (TaKaRa RR01CM). Primer sequences are shown in Supplementary Table S1. PCR products were cloned into the pMD18-T Simple Vector (TaKaRa D103B) and the clones were sequenced. For each region, more than 20 independent top-strand clones were sequenced from each sample. Sequenced results were calculated by using CyMATE (http://www.cymate.org/).

### Accession numbers

Sequence data from this article can be found in the *Arabidopsis* Genome Initiative or GenBank/EMBL databases under the following accession numbers: *MYB74* (At4g05100), *drm1-2* (SALK_031705), *drm2-2* (SALK_150863), *cmt3-11* (SALK_148381), *rdr2-1* (SAIL_1277H08), *dcl3-1* (SALK_005512).

## Results

### *AtMYB74* is induced by salt stress and overexpression lines are hypersensitive to salt stress during seed germination

**Fig. 1.**
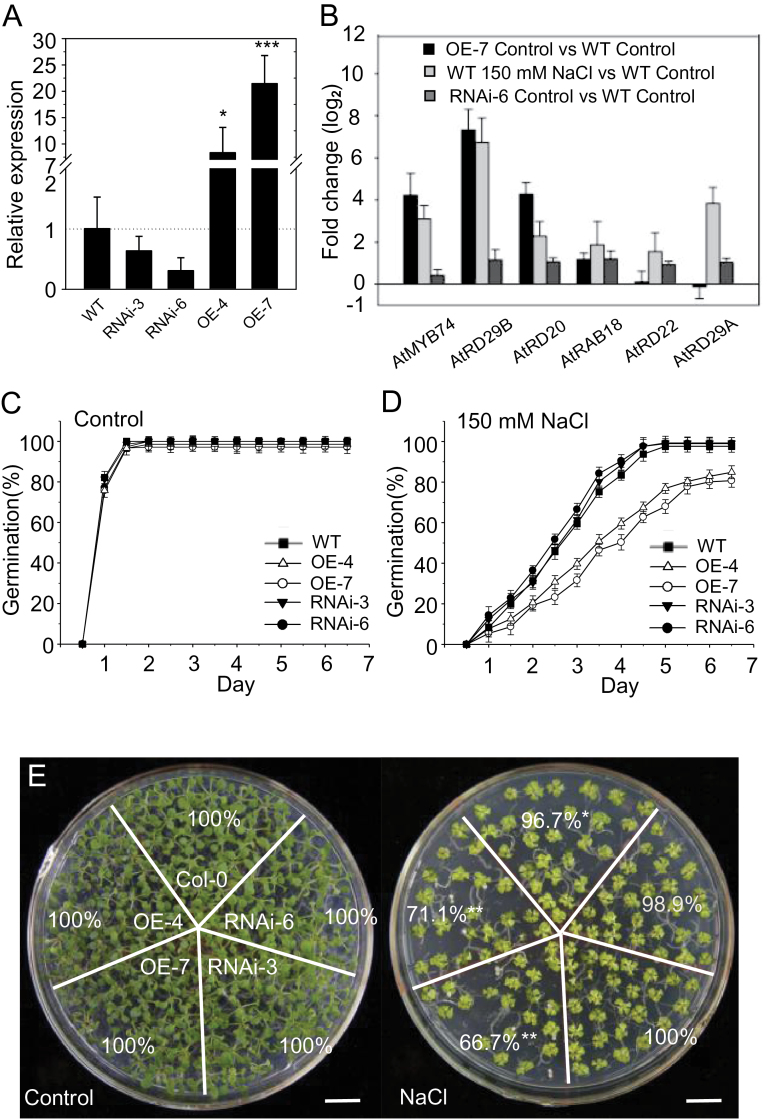
Salt sensitivity of *35S*-*AtMYB74* and RNAi plants during seed germination. (A) Transcript levels of *AtMYB74* in WT, OE and RNAi transgenic lines. Error bars represent SD (*n* = 3). * and *** indicate statistically significant differences at *P* < 0.05 and *P* < 0.001, respectively (Student’s *t*-test). (B) Expression of *AtMYB74* and stress marker genes after treatment with 150mM NaCl for 3h in 14-day-old OE-7 transgenic plants and WT seedlings. Error bars represent SD (*n* = 3). Black bars represent control OE-7 versus control WT; grey bars represent NaCl treatment WT versus control WT. (C, D) Seed germination rates measured on GM agar plates (C) under normal growth conditions and (D) containing 150mM NaCl. Error bars represent SE for three independent experiments. At least 100 seeds per genotype were measured in each replicate. (E) 21-day-old seedlings of WT, OE and RNAi transgenic plants grown on GM agar plates containing 1% sucrose with or without 150mM NaCl. Numbers represent the seedling survival of the different lines. * and ** indicate statistically significant differences at *P* < 0.05 and *P* < 0.01, respectively (Student’s *t*-test). Scale bars = 1cm. (This figure is available in colour at *JXB* online.)

Transcriptome analysis revealed that *AtMYB74* expression level in *Arabidopsis* was increased dramatically by NaCl treatments. To determine the biological function of *AtMYB74* and to test the responses to NaCl, overexpression (OE) and RNAi transgenic *Arabidopsis* lines were generated. In the T3 generation, two independent OE lines and two independent RNAi lines were selected for further experiment (Fig. 1A). Overexpression of *AtMYB74* also induced the expression of a set of known stress marker genes, including *AtRD29B*, *AtRAB18*, and *AtRD20,* all of which contain the conserved MYB recognition sites (TAACTG) in their promoter regions (Fig. 1B). These observations suggest that *AtMYB74* acts as a transcription factor of the salt stress-induced marker genes involved in the salt signalling pathway. Under normal growth conditions, the germination rates of both the OE and RNAi lines were almost the same as those of WT plants, which all achieved rates of 100% at day 2 (Fig. 1C). However, after NaCl treatment, the germination of OE transgenic seeds was inhibited more severely than that of WT seeds, reaching 80% at day 7, when the germination of WT seeds reached almost 100%. The germination rates of RNAi transgenic seeds were similar to those of WT seeds (Fig. 1D). In addition, for 21-day-old seedlings, the percentage seedling survival of OE transgenic lines was much lower than that of WT and RNAi plants under salt stress conditions (Fig. 1E). The reduced germination rates and seedling survival of the OE lines further indicate that *AtMYB*74 is involved in the response to salt stress in plants.

### *AtMYB74* encodes a putative R2R3-MYB transcription factor and is differentially expressed in various tissues

**Fig. 2.**
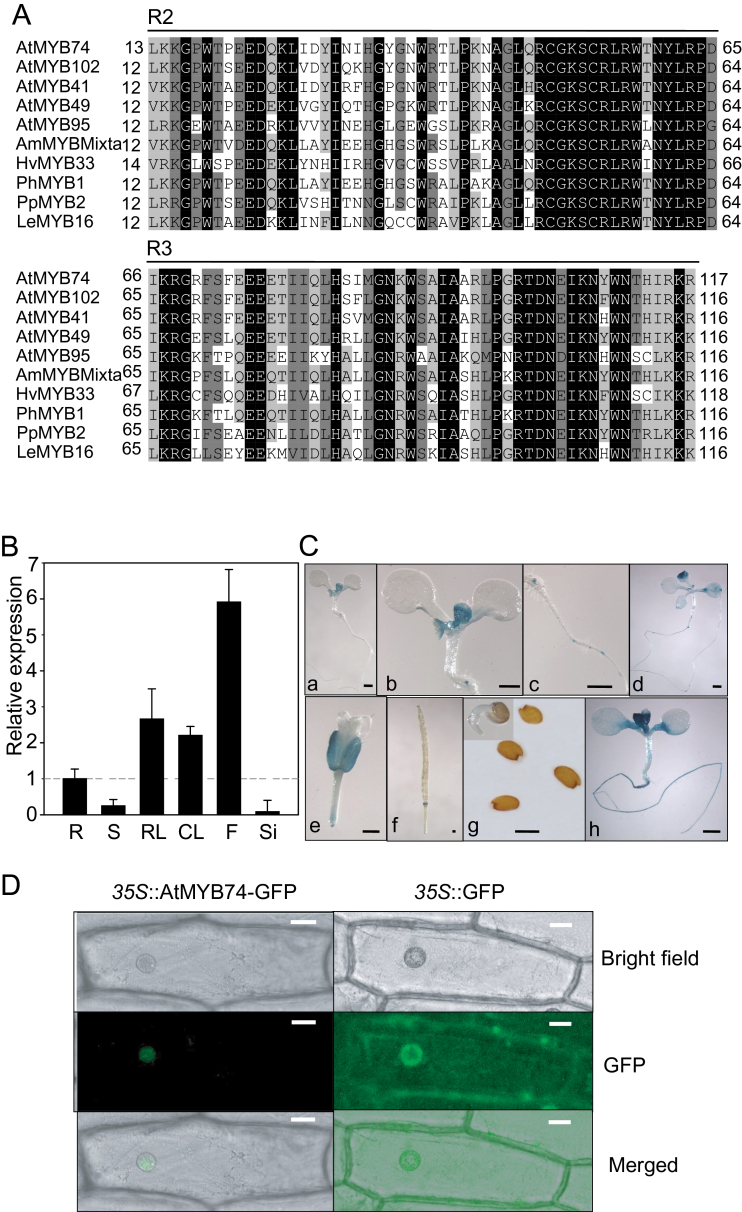
Expression of *AtMYB74* and subcellular localization of AtMYB74 protein. (A) Protein sequence comparison between AtMYB74 and other plant MYB proteins. (B) qRT-PCR analysis of *AtMYB74* expression in various tissues. Results were normalized to the expression of *tubulin*. Error bars represent SD (*n* = 3). R: root, S: stem, RL: rosette leaf, CL: cauline leaf, F: flower, Si: silique. (C) Tissue patterns of a 2kb putative promoter of *AtMYB74*-driven GUS expression in seedlings at different ages or in different tissues: (a) 7-day-old seedling, (b) euphylla, (c) lateral root primordium, (d) 2-week-old seedling, (e) flower, (f) silique, (g) seeds, (h) 7-day-old seedling treated with 150mM NaCl for 3h. Scale bars = 0.5mm. (D) Nuclear localization of AtMYB74 protein in onion epidermal cells. Images in the right column show the control plasmid expressing only GFP and those in the left column show the AtMYB74–GFP fusion protein expressed in onion epidermal cells. The cells were examined with UV fluorescence (top) and bright-field (middle) microscopy, and as a merged image (bottom) showing either the diffuse (control plasmid) or nuclear localization of the proteins. Scale bars = 20 μm. (This figure is available in colour at *JXB* online.)

The full-length cDNA corresponding to the *AtMYB74* mRNA is 975bp in length and encodes a putative protein of 324 amino acids. The R2 and R3 MYB domains (amino acids 13–65 and 66–117, respectively) of AtMYB74 are highly conserved with all other MYB proteins in *Arabidopsis* and in other plant species (Fig. 2A).

To describe the temporal and spatial expression patterns of *AtMYB74* in greater detail, qRT-PCR and promoter–GUS analysis were performed. The highest number of *AtMYB74* transcripts was found in flowers, followed by rosette leaves and cauline leaves, with the roots, stems, and siliques exhibiting the fewest transcripts (Fig. 2B). The tissue pattern of GUS staining was consistent with the qRT-RCR analysis (Fig. 2C). These results suggest that *AtMYB74* is constitutively expressed in various tissues at low abundance.

To detect the subcellular localization of the AtMYB74 protein in plant cells, the *AtMYB74* coding region was fused in the frame to the coding region for the C-terminal side of GFP under the control of the cauliflower mosaic virus *35S* promoter. Onion epidermal cells transformed with an expression plasmid for the AtMYB74–GFP fusion protein exhibited GFP fluorescence in the nucleus (Fig. 2D). However, GFP fluorescence was observed in the entire region of the cell when intact GFP was expressed. These results illustrate that AtMYB74 is localized in the nucleus.

### Dynamic DNA methylation results in *AtMYB74* activation in response to salt stress

**Fig. 3.**
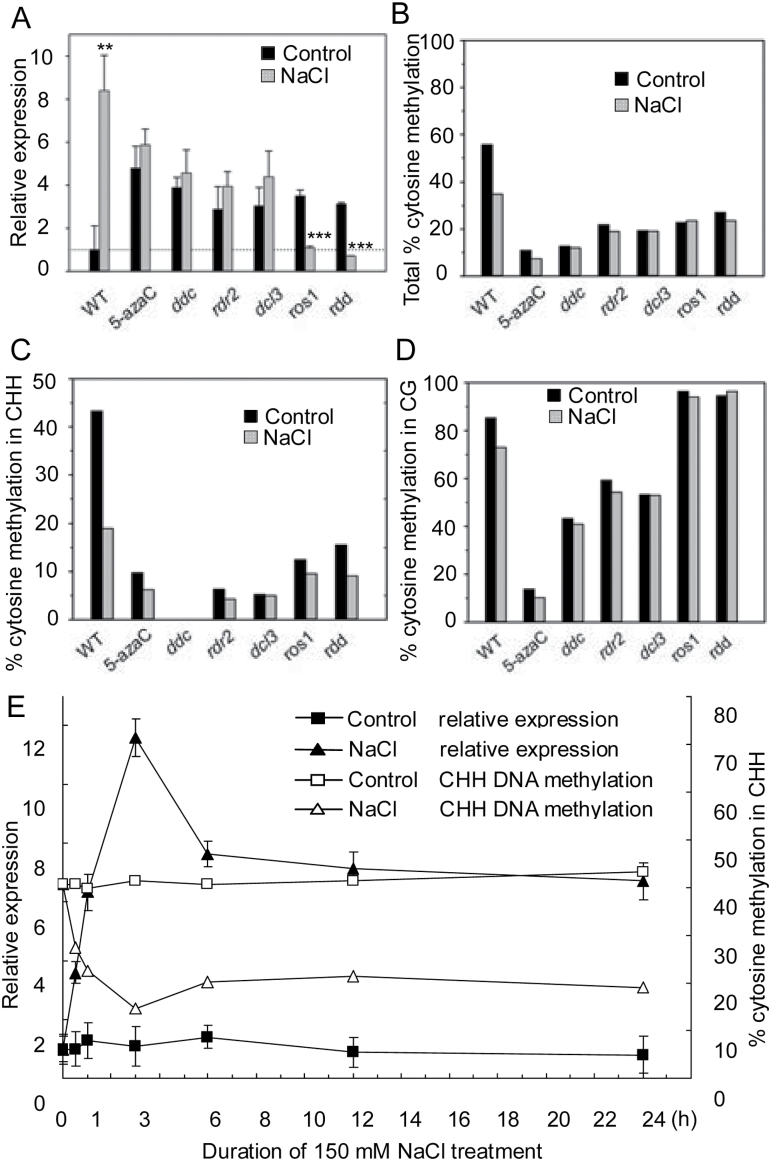
Analysis of *AtMYB74* expression and promoter DNA methylation. (A) Transcript levels of *AtMYB74* in 14-day-old WT, 5-azaC treated WT, *ddc*, *dcl3*, *rdr2*, *ros1*, and *rdd* mutants after 150mM NaCl treatment for 24h. Results were normalized to the expression of *tubulin*. Error bars represent SD (*n* = 3). ** and *** indicate statistically significant differences at *P* < 0.01 and *P* < 0.001, respectively (Student’s *t*-test). (B–D) Bisulphite sequencing analysis of promoter methylation status of *AtMYB74* promoter in 14-day-old seedlings after 150mM NaCl treatment for 24h. Twenty individual clones were sequenced to determine the methylation status of a locus in each genotype. (E) Bisulphite sequencing and qRT-PCR to detect the DNA methylation and expression of *AtMYB74* was at 0, 0.5, 1, 3, 6, 12, and 24h after 150mM NaCl treatment.

Nucleotide sequence analysis revealed that substantial DNA methylation and siRNA target sites exist in the *AtMYB74* promoter region (http://neomorph.salk.edu/epigenome/epigenome.html; http://bioinfo.uni-plovdiv.bg/starpro/). To investigate whether the RdDM pathway regulates *AtMYB74* responses to salt stress, qRT-PCR was used to compare the expression of *AtMYB74* in WT Col-0, 5-azaC (an inhibitor of DNA methylation)-treated WT, *ddc* (*drm1*/*drm2*/*cmt3* triple mutant), *dcl3* (dicer-like 3 mutant), *rdr2* (RNA-dependent RNA polymerase 2 mutant), *ros1-4* (*ros1* mutant), and *rdd* (*ros1/dml2/dml3* triple mutant) in response to 150mM NaCl treatment. As shown in Fig. 3A, the level of *AtMYB74* transcripts increased significantly (~8-fold) in response to NaCl treatment in WT plants, indicating that *AtMYB74* responds to salt stress signals at the transcriptional level. Additionally, the GUS staining assay confirmed that NaCl could enhance *AtMYB74* promoter activity in all tested tissues (Fig. 2C). WT plants treated with 5-azaC, which were used as a control, exhibited a small increase in *AtMYB74* expression under salt stress, indicating the effect of DNA methylation on *AtMYB74* (Fig. 3A). Besides DNA methylation, this result may suggest the presence of other minor factors that may control the expression of *AtMYB74*. For RdDM mutants, the accumulation of *AtMYB74* mRNA in methylation mutants (*ddc, rdr2*, and *dcl3*) showed much less change than that in WT plants under salt stress, whereas the deficiency of active DNA demethylation in *ros1-4* and *rdd* mutants resulted in an obvious decrease in *AtMBY74* expression after salt treatment (Fig. 3A). These results reveal that dynamic DNA methylation results in *AtMYB74* activation in response to salt stress in *Arabidopsis*.

To further understand the mechanism by which RdDM regulates *AtMYB74* expression, the 200bp promoter region approximately 500bp upstream of the transcription initiation site of *AtMYB74* was analysed by bisulphite sequencing. WT plants treated with NaCl exhibited a visible reduction in total 5-methylcytosine content compared with controls (Fig. 3B, Supplementary Fig. S1, Supplementary Fig. S2). Interestingly, the percentage of CHH methylation was nearly halved in the treated WT plants (Fig. 3C), whereas only ~10% reduction in CG contexts was detected (Fig. 3D). No CHG contexts were found in the 200bp region. Moreover, fewer methylated sites in CHH contexts were observed in the RdDM mutants and pharmaceutically treated plants, whether under salt stress or not. Additionally, the three DNA demethylases might not participate in the demethylation of CHH sites in the *AtMYB74* promoter region. The reduction of *AtMYB74* expression in *ros1-4* and *rdd* mutants under salt stress might be regulated by its upstream regulators, which are affected by the demethylases (Fig. 3A, C). These findings suggest that the dynamic balance of DNA methylation and demethylation is crucial for the transcriptional regulation of *AtMYB74.*

Time-course analysis revealed that the level of *AtMYB74* mRNA under salt stress showed a peak from 0.5h, with the highest levels at 3h, accompanied by the lowest percentage of CHH methylation. After 6h, *AtMYB74* expression under salt stress maintained a high level (~7-fold) in treated plants compared with that in the controls, and a lower percentage of CHH methylation was maintained in WT plants (Fig. 3E). These results demonstrate that the increase of *AtMYB74* transcripts was correlated with the lower level of CHH methylation during the NaCl treatment, suggesting that the stress-increased expression of *AtMYB74* resulted from a reduction in dynamic DNA methylation, mainly in the CHH context.

### Decreased DNA methylation is controlled by the level of 24-nt siRNAs targeting the *AtMYB74* promoter

**Fig. 4.**
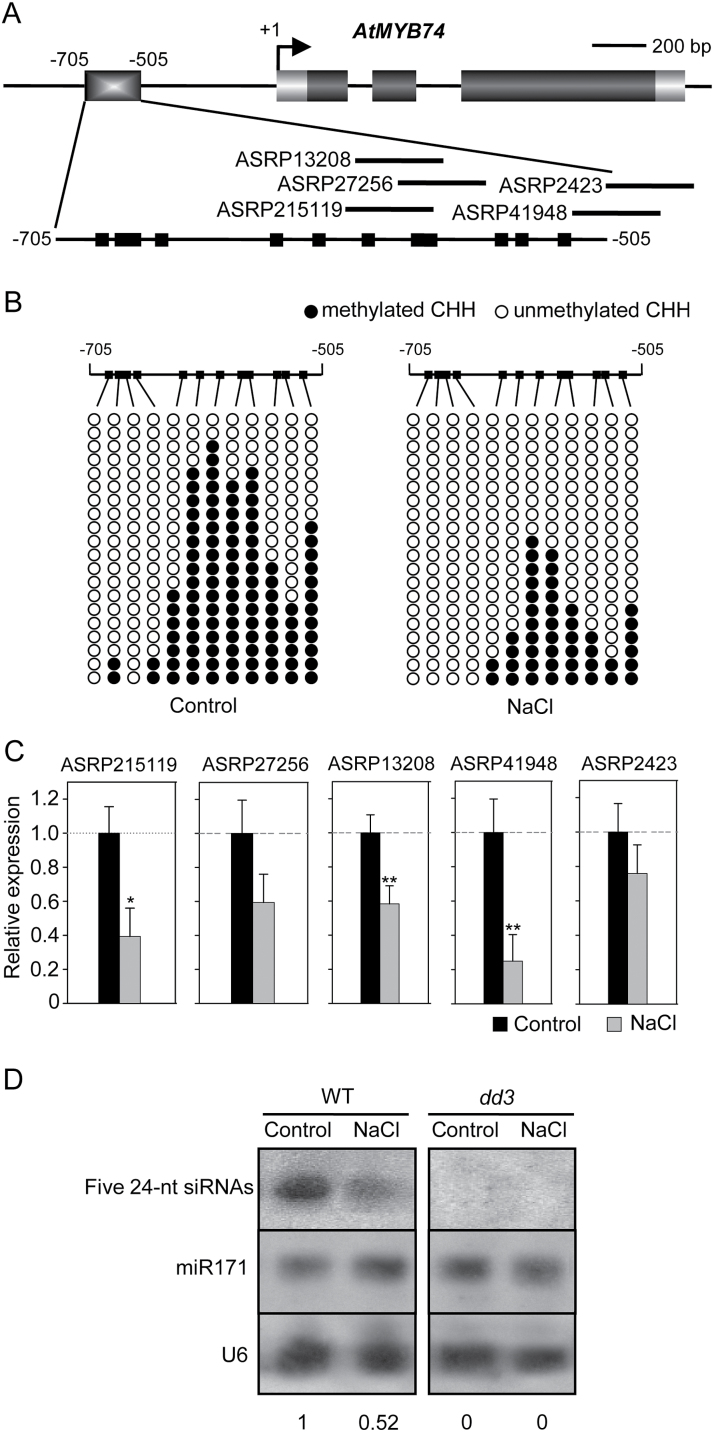
Effect of salt stress on the accumulation of 24-nt siRNAs. (A) Diagram of the *AtMYB74* gene structure; +1 indicates the transcription initiation site. The short lines indicate the five 24-nt siRNAs. Black squares represent CHH configurations. Scale bar = 200bp. (B) Analysis of the cytosine methylation of a 200bp segment spanning the *AtMYB74* promoter in WT plants. Twenty clones per DNA sample were analysed. Filled circles represent methylcytosines in CHH contexts, empty circles represent unmethylated contexts. (C) qRT-PCR analysis of the accumulation of the five 24-nt siRNAs in WT. Results was normalized to the expression of U6. Error bars represent SD (*n* = 3). * and ** indicate statistically significant differences at *P* < 0.05 and *P* < 0.01, respectively (Student’s *t*-test). (D) Northern blot analysis of the NaCl-induced regulation of five 24-nt siRNAs targeting the *AtMYB74* promoter. miR171 and U6 RNA were probed as a control. Numbers under each lane indicate relative expression.

To investigate why CHH hypomethylation occurs in WT plants under salt stress, a sequence analysis of *AtMYB74* was performed using starPRO DB v1.0 (http://bioinfo.uni-plovdiv.bg/starpro/). Five 24-nt siRNAs (ASRP215119, ASRP41948, ASRP27256, ASRP13208, and ASRP2423) were identified to target a narrow region (–603 to –477bp) of the 2.9kb promoter of *AtMYB74*. These five 24-nt siRNAs were located in a cluster in the promoter region (Fig. 4A). Moreover, bisulphite sequencing analysis of individual clones showed that DNA methylation considerably changed in or near the siRNA target region (Fig. 4B). However, no obvious changes were detected in 5-azaC-treated WT or RdDM mutants (Supplementary Fig. S1), indicating that CHH hypomethylation is dependent on RdDM in or near the siRNA target region.

To reveal the role of the siRNAs in RdDM, siRNA qRT-PCR and northern blot analysis were performed. The accumulation of five 24-nt siRNAs was substantially reduced under salt stress in WT plants compared with that in *dcl3* plants (Fig. 4C, D), suggesting that decreases in DNA methylation caused by the reductions in 24-nt siRNA accumulation lead to the activation of *AtMYB74* under salt stress.

### Exogenous 24-nt siRNAs direct RdDM in the *AtMYB74* promoter *in vivo*

**Fig. 5.**
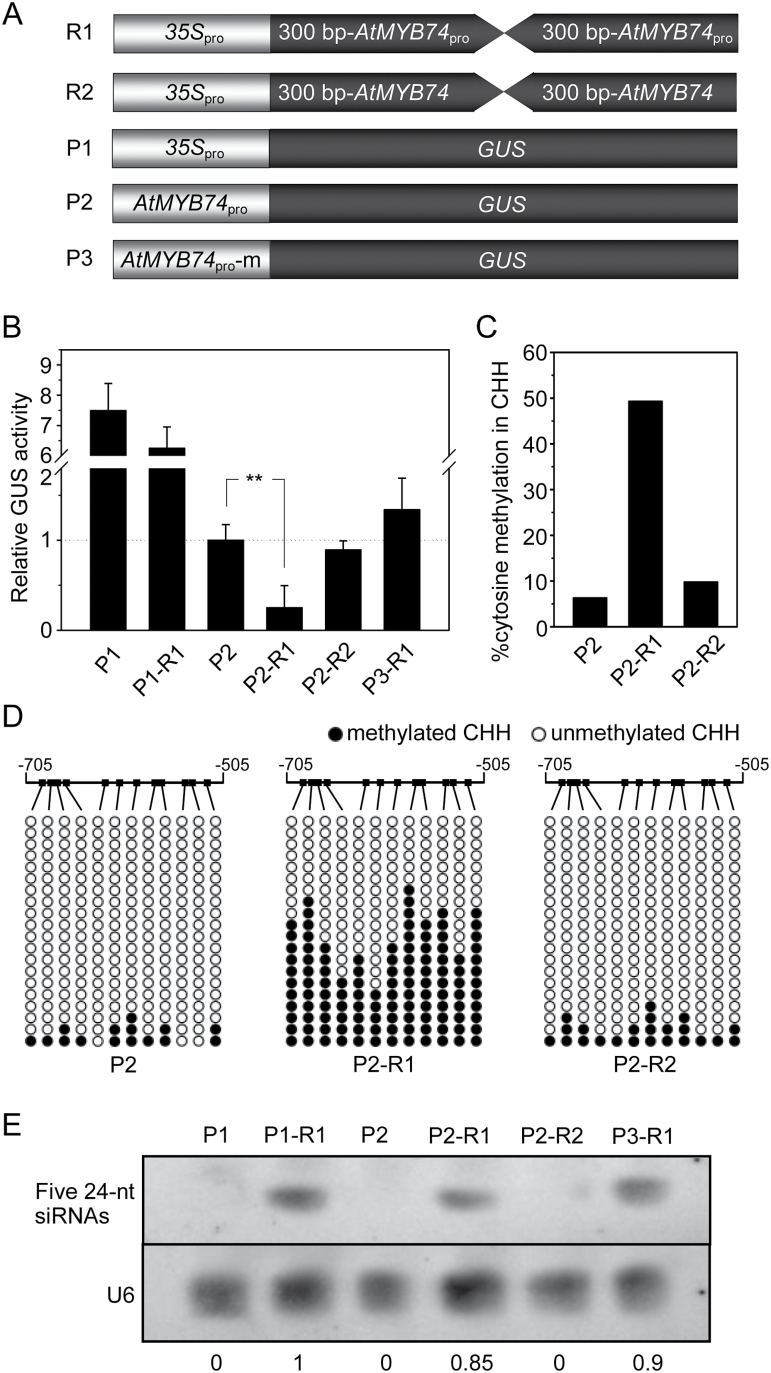
RdDM regulation of the expression of *AtMYB74* in *N. benthamiana*. (A) Schematic diagram of the silencing inducer and the target transgene constructs. The target transgene contains different kinds of *AtMYB74* promoter driven GUS-coding region. The *35S* promoter was used as a control. The hairpin RNA transcribed from the promoter and coding region of *AtMYB74* is diced into silencing RNAs, which induce *de novo* methylation of the target, leading to transcriptional gene silencing of the GUS reporter gene. (B) The effects of siRNAs on GUS expression in transgenic plants. Relative quantification of GUS activity (mean ± SD from three independent experiments) in 3-week-old transgenic *N. benthamiana* leaves. ** indicate statistically significant differences at *P* < 0.01 (Student’s *t*-test). (C, D) Bisulphite sequencing analysis of the three groups containing the 200bp segment spanning the promoter of *AtMYB74*. (E) RNA gel blot analysis of five 24-nt siRNAs targeting the *AtMYB74* promoter in the six groups above. U6 RNA was probed as a control. Numbers under each lane indicate relative expression.

To demonstrate whether the ectopic expression of 24-nt siRNAs affects *AtMYB74* promoter activity, *A. tumefaciens*-mediated transient cotransformation in tobacco (*N. benthamiana*) leaves was performed. Considering that the siRNAs diced from the hairpin RNA could induce transcriptional silencing of the target genes, two types of expression cassettes were constructed to generate siRNAs and express the reporter gene (Fig. 5A). For siRNA expression cassettes in pFGC5941, the 300bp *AtMYB74* promoter containing the siRNA target region that harbors an inverted DNA repeat was used to generate a double-strand RNA as a silencer (R1). A 300bp inverted cDNA sequence of *AtMYB74* was employed as a control (R2). In the reporter gene cassettes of pBI121, *gusA* was driven by the *35S* promoter (P1), *AtMYB74* promoter (P2), and mutated *AtMYB74* promoter with the deletion of a 200bp region where these siRNAs are targeted (P3). As shown in Fig. 5B, GUS activities between groups P1 and P1-R1, P2 and P2-R2, and P2 and P3-R1 did not show obvious changes after transient cotransformation. The GUS activity of group P2-R1 was obviously lower than that of group P2, indicating that promoter activity is affected by the targeted siRNA. In addition, bisulphite sequencing analysis revealed that DNA methylation in the 200bp promoter region increased by 43% due to the siRNAs yielded by the hairpin RNA (Fig. 5C, D). To confirm the accumulation of 24-nt siRNAs in the infection zones of transformed leaves, siRNA northern blot analysis was carried out. The constructs R1, P1-R1, P2-R1, and P3-R1 could efficiently generate siRNAs in the transformed tobacco (Fig. 5E). Overall, these findings demonstrate that the ectopic expression of artificial siRNAs targeting the *AtMYB74* promoter also can regulate GUS expression through RdDM.

## Discussion

RdDM, which controls the expression of a number of genes in many developmental processes and abiotic stress responses, has been considered an important epigenetic pathway (Borsani *et al.*, 2005; Chan *et al.*, 2006; Henderson and Jacobsen, 2008; Saze and Kakutani, 2007). However, only a few transcription factors regulated by RdDM, such as *b1* and *C-LEC1*, have been identified (Alleman *et al.*, 2006; Shibukawa *et al.*, 2009). Although many members of the R2R3-MYB family, such as *AtMYB2*, *AtMYB108*, *AtMYB41*, and *AtMYB102*, are reported to be involved in abiotic stress responses in plants (Abe *et al.*, 2003; Denekamp and Smeekens, 2003; Lippold *et al.*, 2009; Mengiste *et al.*, 2003), little is known about whether and how RdDM participates in this response pathway. The present study characterized *AtMYB74*, a putative R2R3-MYB member. DNA methylation of the promoter of *AtMYB74* negatively correlates with gene expression under salt stress. Moreover, when the RdDM region is deleted from the *AtMYB74* promoter, transgenic plants display higher GUS activity, suggesting that RdDM as a repressive epigenetic modification affects transcription of *AtMYB74* (Fig. 5). Using transgenic *Arabidopsis* overexpressing siRNAs (R1) further confirmed this conclusion (Fig. S3). Overall, this study demonstrates that RdDM directly controls the expression of *AtMYB74*, providing a novel regulatory pathway in the R2R3-MYB family. Using a gain-of-function approach, and according to the phenotypes of *AtMYB74* overexpressing and RNAi transgenic plants, the findings suggest that there may be functional redundancy of the R2R3-MYB family, as reported by Millar and Gubler (2005). These results may point to the existence of a network of inter-regulated MYB genes, including *AtMYB2* and other members of the subgroup of *AtMYB74,* that respond to abiotic stresses in plants.

Previous studies have indicated that siRNAs and long non-coding RNAs could be involved in *de novo* DNA methylation (Wierzbicki *et al.*, 2008). Comparison of genome-wide methylation patterns and small RNAs in *Arabidopsis* reveals that ~37% of the methylated loci are related to siRNA clusters (Schmitz *et al.*, 2011; Zhang *et al.*, 2006), suggesting a close relationship between DNA methylation and siRNAs. The present study identified a cluster of siRNAs that directly mediates DNA methylation in or near the target region of *AtMYB74* in *Arabidopsis*. Transformation in tobacco using artificial siRNAs yielded by hairpin RNA further demonstrated the involvement of RdDM in the native siRNA-targeted region. Interestingly, artificial siRNAs can also direct DNA methylation in the 200bp region, separately from the native siRNA-targeted region (Fig. 5), implying that any artificial siRNA may regulate promoter activity via DNA methylation in its target region. The accumulation of 24-nt siRNAs and changes in CHH DNA methylation have similar tendencies in *Arabidopsis* under salt stress, suggesting that the accumulation of 24-nt siRNAs is the trigger of RdDM, in agreement with a previous study (Melnyk *et al.*, 2011). In rice, it was reported that siRNAs generated by *OsRDR6* regulate the post-transcriptional gene silencing of the isocitrate lyase (ICL) gene (Yang *et al.*, 2008). Taken together, the present observations reveal that the RdDM component may affect the accumulation of 24-nt siRNAs and then trigger DNA methylation of *AtMYB74* under salt stress, providing strong evidence of epigenetic modification in the plant response to abiotic stress. Further work is necessary to determine the mechanisms of RdDM components that affect the accumulation of 24-nt siRNAs and the biochemical function of *AtMYB74* in response to salt stress.

**Fig. 6.**
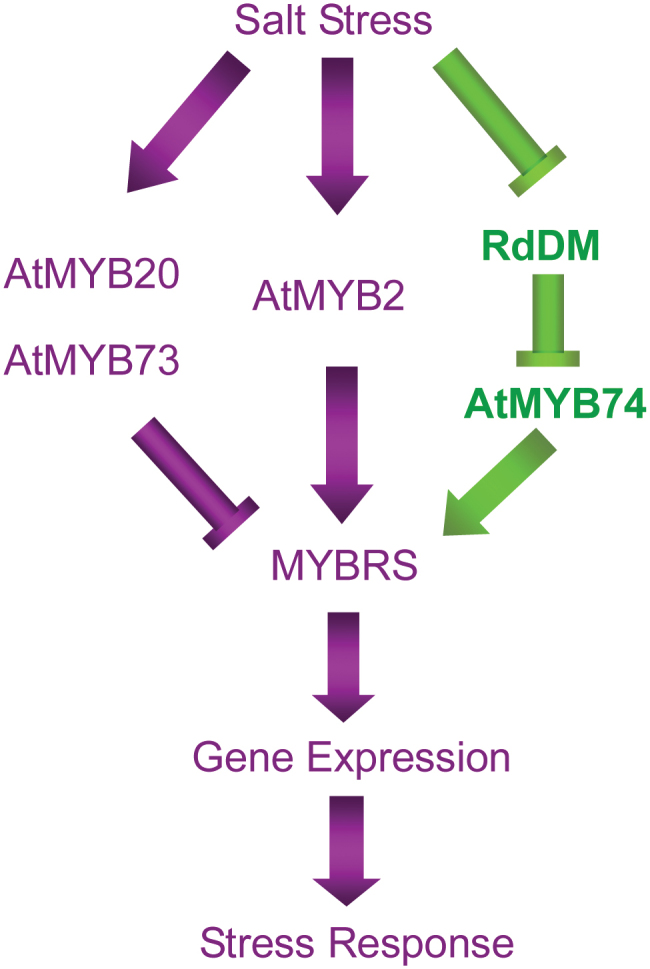
Schematic model of the transcriptional regulation of salt stress signalling via MYB, showing the involvement of a putative R2R3-MYB transcription factor member, *AtMYB74*, regulated by RdDM, in controlling the positive regulation of transcriptional responses to salt stress. The purple arrow represents results obtained from previous studies, and the green arrow represents the results of the present study. (This figure is available in colour at *JXB* online.)

The salt stress signal transduction pathway is far more complex than has been suggested previously. As shown in Fig 6, previous studies have shown that some R2R3-MYB transcription factors, such as AtMYB2, AtMYB20, and AtMYB73, participate in the regulation of gene expression under salt stress (Abe *et al.*, 2003; Cui *et al.*, 2013; Kim *et al.*, 2013). The present study demonstrated the involvement of *AtMYB74*, a R2R3-MYB family member, which is regulated by RdDM in controlling the positive regulation of transcriptional responses to salt stress. Salt stress induces the RdDM pathway via repressing the accumulation of siRNAs to activate *AtMYB74* expression, which then transduces the related signalling into downstream processes that endow resistance to such stress.

## Supplementary data

Supplementary data are available at *JXB* online.

Supplementary Fig. S1. Bisulphite sequencing analysis of promoter methylation status of *AtMYB74* promoter in 14-day-old 5-azaC treated WT, *ddc*, *dcl3*, *rdr2*, *ros1-4*, and *rdd* mutants after 150mM NaCl treatment.

Supplementary Fig. S2. A schematic of the *AtMYB74* promoter sequence with bisulphite sequencing region.

Supplementary Fig. S3. RdDM regulation of *AtMYB74* expression in transgenic *Arabidopsis*.

Supplementary Table S1. Primers and probes used in this study.

Supplementary Data

## Abbreviations:

5-azaC: 5-azacytidine
CBF: CCAAT-binding transcription factor
CMT3: chromomethylase 3
DCL3: dicer-like 3
ddc: *drm1/drm2/cmt3* triple mutant
DML: DEMETER-like protein
DRM: domains rearranged methyltransferase
GUS: β-glucuronidase
MET1: methyltransferase 1
MS: Murashige and Skoog medium
NAC: NAM, ATAF1/2, CUC2 transcription factor
NRP: nuclear RNA polymerase
OE: overexpression
qRT-PCR: quantitative real-time reverse transcription PCR
rdd: *ros1/dml2/dml3* triple mutant
RdDM: RNA-directed DNA methylation
RDR2: RNA-dependent RNA polymerase 2
ROSl: repressor of silencing 1
rpm: revolutions per minute
TGS: transcriptional gene silencing
WT: wild type.
