# Supplementary material for: Salt-induced transcription factor MYB74 is regulated by the RNA-directed DNA methylation pathway in Arabidopsis
Source: J Exp Bot. 2015 Jul 2;66(19):5997–6008. doi: 10.1093/jxb/erv312 (PMC4566987; doi:10.1093/jxb/erv312)
Supplement: Supplementary Data [file supp_66_19_5997__index.html]

Salt-induced transcription factor MYB74 is regulated by the RNA-directed DNA methylation pathway in Arabidopsis — Salt-induced transcription factor MYB74 is regulated by the RNA-directed DNA methylation pathway in Arabidopsis — Supplementary Data 

# Salt-induced transcription factor *MYB74* is regulated by the RNA-directed DNA methylation pathway in *Arabidopsis*

## Supplementary Data

Data files

- Supplementary Data - Supplementary Data
