# Supplementary material for: Salt-induced transcription factor MYB74 is regulated by the RNA-directed DNA methylation pathway in Arabidopsis
Source: J Exp Bot. 2015 Jul 2;66(19):5997–6008. doi: 10.1093/jxb/erv312 (PMC4566987; doi:10.1093/jxb/erv312)
Supplement: Supplementary Data [file supp_erv312_jexbot148619_file001.pdf]

Figure S1

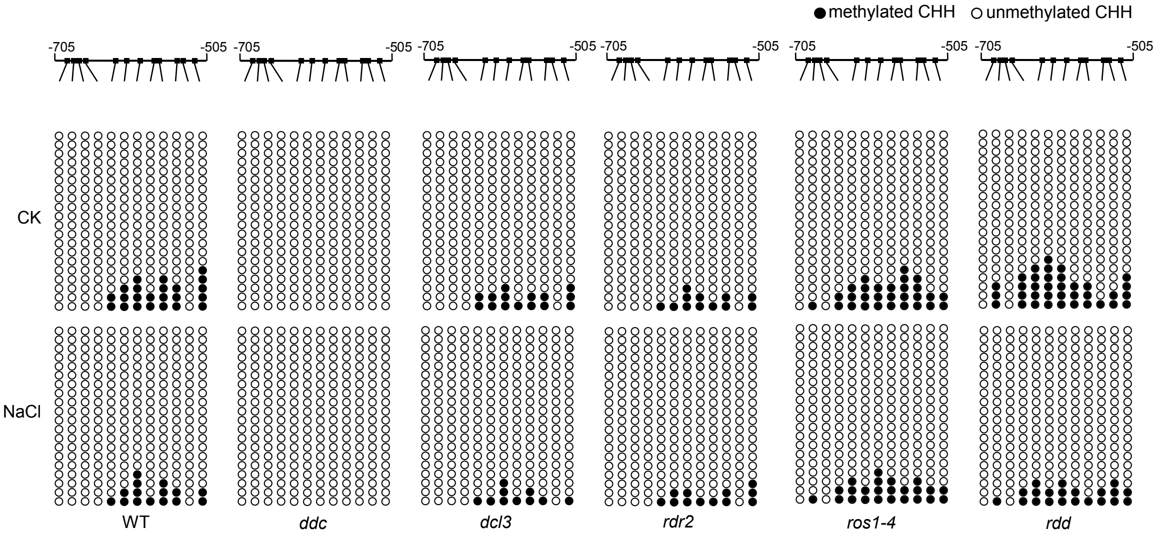

Figure S1. Bisulfite sequencing analysis of promoter methylation status of *AtMYB74* promoter in 14-day-old 5-azaC treated WT, *ddc*, *dcl3*, *rdr2*, *ros1-4*, and *rdd* mutants after 150 mM NaCl treatment. The CK (control) use H_2O instead.

tttttagaaaaattattgattgtagagaggaactaccttttggattaaatttttgtaaaaaattacaaatttttttaaaatctctccataa
attaataattattaatttatcgataaattaatacctctctaaattaataaatttttgtactattattacctagaaagtgaaaattgacct
acaaatatatcttcaatttgattcacatatacgaagtcacgtatgtttctataaatttagatccaaaatgtgtgaaaacccatctgtt
tttgcaaattcttgcaattgcaattgcacatgtttctattacgtttaaatcaatcgagctctaagtgatttatccatagtgatatacag
atccaatatccatgaaacatatttaattcatgaaaaggctcaaacattatcaacaaataagatatggtcaactatgtagtaact
agtaacacgtaggctgctgctctttgtggcaaattcatgcgcaagcaaagcctctagatgtttgacaagttactaccctaatata
gcatcactagctatcttctaatttttttttttagttattttaatcctgtattcttttatttatttatttgataaagtgatgaaattttattctccttttt
cttatcaactaatatacttattcattcacataatttcgcactttgcggagttttcatttgtcgttaaacccgacagattcttgaaccgc
aaggaacgagacattaacctaatgtacttatatttaactatagtattataatagcataaatgtgacaatgattcgatcactacta
atatattaatcaccaatctatatcgtcgtccttcctgattatttgaagattttgtctataaactagctgcttttgtttatgtatttttattactt
ggatgcttttgtttgttggaatattcgaatgaatttttgtttgcctgaaactatgcatatataataaggccatatgcgccaataatata
gaatccaaatattcattaactataattagtttgttctagagttttagctactcatatattcaaattcttaggagatcaaaaaagtactt
aattagttaataaaatccatttttattagttgtaattaaatgtaaagtgtcgaaacacgaaagggaagatgagttgcacaactcg
tggttcctataagagtgataatctatctgacgtcacgccatgcaaaatgtatcaatcaattttcttctcacatttatatttacttcaaa
atgattataaagccacccaatgaataataagatatatatatatatatatataactcaaaatcaaaaatttcaaatggaatttgga
agcatcacaaattcacaatcatgtattcaaaatctcacgtagaaaaagctaataagtccaaaagattggtcttcatttttttcttg
agaaagtttgatgggatagagagagagacagagaggaacagctaaatttgctgtgtatgtatttaatacttgaacatttatttat
ctattttcttatacatcaaactcacgaatttataggttggtaatgtcgtttttataacattaataaaaattgcaattgtagtttttattgaa
atttacccaaaaaaaactattatacgaaaatatgttatttatttaaaatttgatattagatgaatccagaatcttttataaaacttggt
gtgtgtgaacctgcgttaatttgttaaatacttttcacgtaaaaatcaaaattaattttaaaagttatgttaccaaacagtattctgat
agttttacaaattttatccattgtatatgttttgtttttgcaaaaaagtccaaaggaaaaaacaaagcagaagggttgaattgtga
ggaaagagagagtacgtggcatcataactttggcgacagctaatgtgaaaacagccaaaagctacggagagcgacact
cctcgacgacgtccatgtctctttagattgtgattgcattgttaattatcatatttatcaattgattagttaagtcttaagtatcatcaca
caagtttatacattaatctatacagtgtttaacactacaaaactccccaaaatcacgtgtttcatatcatacgtacatttgtctacc
gtctttattaacaatattaaaacagaatttatcaattttctagttgaatagactatatactattatagagactcttataaattatgaaa
ctcctaatcaattaaaaatagttaatttgaaacatatcactcgaaactagggtattagttggattgacgatatttttcttattaatatc
aacgagaagaatcaagtgtcgttagctcaattggtaaaaaatttaggcaaagcttagagatgtctatttgagtgacgcttgga
acaaaaattacatgctatggtttcaggcctagagaaatgcttcgatccagaatctcttgatattcaaaaaaaaaaaaaaaatc
aacaagaagaatgtataaatggtttctatactatctaagttatgaaaaatatttttggttacccaaattacaagatttgtaccgaa
gtgtcacactagattcacatgcatgcatagttataattaacttaaaacataaatgacttacaaaaatacttacaaacaaaaac
caaattaagttatttagtggccagctttgttactagagttttagccattatcataattcacaataaggtgtaggaaaaaacattaat
atatagtatgtatacatccaaaaaaaacttttggaaaaaacaaattttgggattataaaggacacgtagcccaatcgatgaa
cagaggacggcaccgaatcaatttgacttcacctttttccccatatgataagagtctcatctctttcctatatatatttcgtacctct
ctttaaggaccctacgtctctctctttctctctactctctgtttcctcataattcaatcactatattttttaaaaacatttgacttcatcgat
cggttaacaattaatcaaaaagATG

Figure S2. A Schematic of *AtMYB74* promoter sequence with bisulfite sequencing region. The bisulfite sequencing region is shaded in red. The yellow shaded sequence is 5′ UTR region.

Figure S3

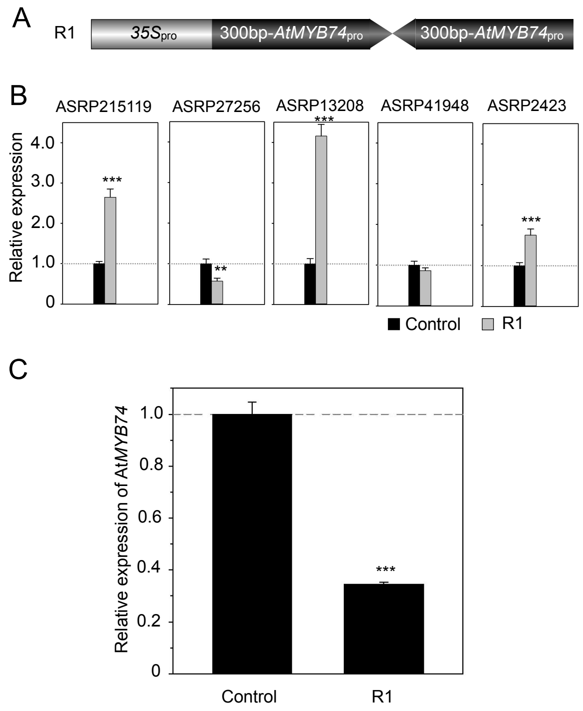

Figure S3. The RdDM regulation of *AtMYB74* expression in transgenic *Arabidopsis*

A A schematic diagram of the silencing inducer construct. The hairpin RNAs transcribed from the promoter of *AtMYB74* are diced into siRNAs, which induce *de novo* methylation of the target leading to transcriptional gene silencing of the native *AtMYB74* gene. **B** qRT-PCR analysis of the accumulation of the five 24-nt siRNAs in WT(Control) and R1 transgenic lines. Results was normalized to the expression of U6. Error bars represent SD (n = 3). ** and *** indicate statistically significant differences at $P < 0.01$ and $P < 0.001$ (Student's *t*-test). **C** Transcript levels of *AtMYB74* in WT and R1 transgenic lines. *** indicate statistically significant differences at $P < 0.001$ (Student's *t*-test).

Table S1. Primers and probes used in this study.

Primer	Purpose	Sequence*
P-MYB74-5′	vector	ggtacctcgtcgtccttcctgattattt
P-MYB74-3′	vector	ggatccgaggaaacagagagtagagagaa
PD-MYB74-5′	vector	ggatccgcggtcgaccctagtttcgagtgatatgtttc
PD-MYB74-3′	vector	gtcgaccaaattacaagatttgtaccgaa
cDNA-MYB74-5′	vector	ggatccctctctactctctgtttcctcata
cDNA-MYB74-3′	vector	gtcgacactctattccaatggcgtttt
MYB74-GFP-5′	vector	tctagaggatccctctctactctctgtttcctcata
MYB74-GFP-3′	vector	gtcgacttggaattggagaaaaccattaaca
RNAi-5′	vector	tctagaggcgcgccgtggaccaactatctccgacc
RNAi-3′	vector	ggatccatttaaatgatgatgatgatgatgcgaagag
P-RNAi-5′	vector	tctagaggcgcgccattatgaaactcctaatcaat
P-RNAi-3′	vector	ggatccatttaaataaatcttgtaatttgggtaacca
BS-5′	BSP	ataaaaaaacaaaaaataaaaaaaa
BS-3′	BSP	tattattataatttataataaggtgtagga
ASRP215119-5′	qRT-PCR	gcgagctaacgacacttgattcttct
ASRP41948-5′	qRT-PCR	gcaggcctgaaaccatagcatgtaat
ASRP27256-5′	qRT-PCR	cgctttttaccaattgagctaacgaca
ASRP13208-5′	qRT-PCR	gcaccaattgagctaacgacacttga
ASRP2423-5′	qRT-PCR	gcgatcgaagcatttctctaggcctg
U6-5′	qRT-PCR	tggcccctgcgcaaggatga
Tubulin 5′	qRT-PCR	acggacgctacctcacag
Tubulin 3′	qRT-PCR	agtaggacgagttcttgttctg
MYB74-5′	qRT-PCR	agaacagacaacgagat
MYB74-3′	qRT-PCR	gatgatgatgcgaagag
Total siRNA-5′	northern blot	gaaacatatcactcgaaactagg
Total siRNA-3′	northern blot	tgtgacacttcggtacaaatctt
miR171	northern blot	gatattggcgcggctcaatca
U6	northern blot	tcatccttgcgcaggggcca

***small RNA qRT-PCR 3′ primers used in mature miRNA qRT-PCR assay were acquired from miRcute miRNA qPCR Detection kit.**
